# Commentary: Novelty seeking and reward dependence-related large-scale brain networks functional connectivity variation during salience expectancy

**DOI:** 10.3389/fpsyg.2018.00242

**Published:** 2018-02-27

**Authors:** Cristiano Crescentini

**Affiliations:** Department of Languages and Literatures, Communication, Education and Society, University of Udine, Udine, Italy

**Keywords:** personality, temperament, character, executive functions, salience, brain networks

In recent years several neuropsychological and psychiatry studies employed the psychobiological model of temperament and character (TCI; Cloninger et al., 1993) to investigate the relationship between personality and neuropsychological function in patients with Parkinson's disease (Koerts et al., 2013), Friedreich Ataxia (Sayah et al., 2017), attention-deficit/hyperactivity disorder (ADHD; Drechsler et al., 2015), eating disorders (Pignatti and Bernasconi, 2013), and antisocial behavior (Bergvall et al., 2003). These studies indicated that alterations in personality and cognition are not independent from each other, in that poor development of specific personality traits appears to be associated with deficits in neuropsychological performance, in particular in advanced cognition such as executive functions (EF: attention, working memory, planning, set-shifting, inhibition).

Recently, a great attention has been paid to salience as a fundamental component and modulating factor of attention regulation (Uddin, 2015). It has been argued that the dorsal anterior cingulate cortex (dACC) and the anterior insula (AI) constitute a salience network (SN). The SN would be involved in the attentional control function of detecting subjectively salient events and would provide control signals to a central-executive network (CEN, including the dorsolateral prefrontal cortex and the posterior parietal cortex) to act upon in agreement with current goals (Menon, 2015; Uddin, 2015). Tellingly, a few past studies have associated salience-related internal expectancy behaviors (which employ top-down goal-directed attention regulation) with TCI novelty-seeking (NS) and harm-avoidance (HA) temperaments (Most et al., 2006; Bermpohl et al., 2008; Zhang et al., 2017).

In a recent study, Li et al. (2017) have provided crucial neuroimaging (fMRI) evidence about how functional connectivity (FC) and activation within and beyond the SN can be modulated by internal salience expectancy and temperament. Li et al. explored salience-related connectivity changes (using psychophysiological interaction analysis, PPI) during the anticipation periods involved in a salience expectancy task, in which a group of healthy adults (*n* = 68) had to rely on visual cues (arrows pointing up or down) in order to actively expect the high or low salience of the following pictures (positive and neutral pictures). Furthermore, correlations between PPI maps and temperamental traits were ran, focusing specifically on NS (exploratory/impulsive vs. indifferent/reflective), HA (worrying/anxious vs. relaxed/confident), and RD (reward dependence: sentimental/dependent vs. practical/independent) TCI temperaments.

Critically, concerning FC, Li et al. found that both the right dACC and the right AI (the two areas used as PPI seed regions) showed positive FC with parts of the CEN and negative FC with posterior visual areas and parts of the default mode network (DMN) as a function of high versus low salience expectancy. Moreover, with regard to FC and personality correlation, Li et al. (2017) found reduced FC between right AI and right middle cingulate cortex (MCC) with increasing NS, and reduced FC between right dACC and caudate, with increasing RD. These correlations occurred when participants were expecting high-salience pictures as compared to low-salience pictures, suggesting that whether participants had high or low NS and high or low RD led them to use different salience expectancy processing neurocognitive mechanisms.

As argued by Li et al., the findings confirmed that the SN is important for internal salience evaluation and for the control of goal-directed behavior; more critically, they showed that SN activity, and its integration with other brain networks during salience processing, can be modulated by specific temperamental traits.

The study by Li et al. (2017) thus shed further light on the relation between personality and neuropsychological function and has important clinical implications. Considering past evidence showing associations between temperament and risk of addiction (see Crescentini et al., 2015 for a brief review), the authors argued that addiction behavior may result in part from structural or functional impairments of the SN and associated affective/reward systems (e.g., MCC, caudate), which may lead to dysfunctions during salience expectancy (e.g., perceiving low-salience stimuli with higher significance) depending on individuals' personality predispositions. This possibility is in line with the conclusion of Uddin (2015) suggesting that atypical engagement of the SN by subjectively salient stimuli, together with atypical patterns of FC with other brain networks (CEN, DMN), could lead to dysfunctions of salience and attentional processing that are characteristic of many neuropsychiatric disorders, among which are schizophrenia and autism.

In their study, Li et al. used only positive and neutral pictures in the salience expectancy task, and only temperament traits were put into relation with salience processing and SN function. Hence, it would be interesting to see in future studies whether using more stressful stimuli in similar expectancy tasks (e.g., negative picture) discloses the mediating role of other temperaments (e.g., HA). For example, past research has shown that HA affects behavior and brain functions when negative emotional stimuli are used in visual attention tasks, especially under conditions of uncertain expectations (Most et al., 2006). More critically, it would be important to extend the current findings to the other major component of personality, namely the character. First of all, this will foster our knowledge on the association between character and neuropsychological functions related to salience and attention. In this regard, a recent investigation on healthy adults has shown that high Self-Directedness (SD: purposeful/responsible/reliable vs. purposeless/blaming/unreliable), a crucial aspect of character maturity in the TCI, is protective against distraction by highly salient picture stimuli during an audiovisual attentional conflict task, a finding suggestive of better goal-directed behavior in individuals with higher, vs. lower, SD (Dinica et al., 2015). Furthermore, future research on the relation between personality and neuropsychology could inform clinical applications on how best to assess and improve goal-directed behavior and high-level cognitive control functions. Indeed, this could require focusing directly on these functions but also attempting to enhance individuals' character acting on aspects such as maturity, autonomy, perseverance, purposefulness, self-regulation, and self-acceptance (Diamond and Lee, 2011). Crucially, in several clinical researches it was shown that character maturity may indeed have a protective role against persistent manifestation of negative outcomes in individuals with antisocial behavior, conduct disorder, ADHD, and personality disorders (Svrakic et al., 1993; Bergvall et al., 2003; Drechsler et al., 2015; Gomez et al., 2017; Kerekes et al., 2017).

## Author contributions

The author confirms being the sole contributor of this work and approved it for publication.

### Conflict of interest statement

The author declares that the research was conducted in the absence of any commercial or financial relationships that could be construed as a potential conflict of interest.
